# Decreased Plasma Levels of Growth Differentiation Factor 11 in Patients With Schizophrenia: Correlation With Psychopathology and Cognition

**DOI:** 10.3389/fpsyt.2020.555133

**Published:** 2020-12-07

**Authors:** Zhao-xi Yang, Jin-qiong Zhan, Jian-wen Xiong, Bo Wei, Yong-hui Fu, Zhi-peng Liu, Ya-ting Tu, Yuan-jian Yang, Ai-lan Wan

**Affiliations:** ^1^Department of Psychosomatic Medicine, The First Affiliated Hospital of Nanchang University, Nanchang, China; ^2^Biological Psychiatry Laboratory, Jiangxi Mental Hospital/Affiliated Mental Hospital of Nanchang University, Nanchang, China; ^3^Department of Psychiatry, Jiangxi Mental Hospital/Affiliated Mental Hospital of Nanchang University, Nanchang, China; ^4^Jiangxi Provincial Clinical Research Center on Mental Disorders, Nanchang, China

**Keywords:** schizophrenia, psychopathology, cognition, growth differentiation factor 11 (GDF-11), correlation

## Abstract

Schizophrenia is linked with abnormal neurodevelopment, on which growth differentiation factor 11 (GDF-11) has a great impact. However, a direct evidence linking GDF-11 to the pathophysiology of schizophrenia is still lacking. The current study aimed to investigate the relationship between plasma GDF-11 levels and both psychopathological symptoms and cognitive function in schizophrenia. Eighty-seven schizophrenia patients and 76 healthy controls were enrolled in the present study. The symptomatology of schizophrenia was evaluated using the Positive and Negative Syndrome Scale (PANSS). Cognitive function was assessed by Repeatable Battery for the Assessment of Neuropsychological Status (RBANS) including twelve neurocognitive tests in five aspects of cognitive function. Plasma GDF-11 levels were determined by enzyme-linked immunosorbent assay (ELISA). We found that plasma levels of GDF-11 were significantly lower in schizophrenia patients relative to healthy controls. Correlation analysis showed significant negative correlations between the GDF-11 levels and the PANSS total score, the positive symptoms score, the negative symptoms score or the general score. Additionally, positive associations were observed between plasma GDF-11 levels and the visuospatial/constructional, attention, immediate memory, or delayed memory in patients. Partial correlation analysis showed that these correlations were still significant after adjusting for age, gender, education years, body mass index, duration of illness, and age of onset except for the visuospatial/constructional and attention index. Multiple regression analysis revealed that GDF-11 was an independent contributor to the immediate memory, delayed memory and RBANS total score in patients. Collectively, the correlations between plasma GDF-11 and psychopathological and cognitive symptoms suggest that abnormal GDF-11 signaling might contribute to schizophrenic psychopathology and cognitive impairments and GDF-11 could be a potential and promising biomarker for schizophrenia.

## Introduction

The neurodevelopmental hypothesis has proposed that the abnormalities of brain development are involved in the etiology and pathogenesis in schizophrenia (1, 2). Clinical, epidemiological, genetic and neuropathological studies performed in schizophrenia have provided numerous evidence for supporting this hypothesis (3, 4). For example, the brain gray matter and volume of children with schizophrenia are smaller than those of healthy children, and the decrease of gray matter density is earlier than that of schizophrenia (5). The abnormal brain development of patients with schizophrenia has occurred in the fetal stage, and this pathological change persists through childhood (6). A 45-year demographic follow-up survey in Denmark also reveals that patients with schizophrenia tend to lag behind their peers in its growth and development, even in the first year after birth (7).

Growth differentiation factor 11 (GDF-11) is a secretory protein of the transforming growth factor beta (TGF-β) superfamily that regulates diverse cellular processes. GDF-11 has a critical impact on development, including anterior/posterior patterning (8), kidney formation, spleen, stomach and endocrine pancreas (9, 10). Recent studies have demonstrated that GDF-11 has a regulatory role in the formation of the central nervous system (CNS). GDF-11 is intensely expressed in astrocytes, ependymal cells, neurons and their axons (11). During the development period, GDF-11 is able to modulate the patterning of CNS and the genesis, differentiation, maturation, and activity of new brain cells (8, 12). In addition, high content of plasma GDF-11 is observed when the body is young and it decreases gradually with aging (13, 14). Systemic GDF-11 treatment in rodents could improve vasculature in the cortex and hippocampus (15).

Recent studies have suggested that GDF-11 might be implicated in the pathogenesis of schizophrenia. Glutamate imbalance is an important cause of neuroinflammation (16, 17). Activation of astrocytes can directly affect the glutamate concentration in the synaptic gap (18), thus the changes in neuroinflammation caused by activation of astrocytes might contribute to the pathogenesis of schizophrenia. Furthermore, GDF-11 can modulate the function of astrocytes by regulating the expression of glutamate dehydrogenase (11). Lander et al. showed that mice lacking glutamate dehydrogenase (GDH), a glutamate-metabolizing enzyme, displayed schizophrenia-like psychotic symptoms and cognitive disorders, as well as hippocampal dysfunction (19). Cognitive impairments are considered as a core characteristic of schizophrenia. Treatment with GDF-11 in aging mice improved olfactory discrimination via promoting the cerebral vasculature and enhancing neurogenesis (20). A single dose of recombinant GDF-11 increased hippocampal transcription factor Sox2 expression and promoted short-term spatial and visual memory in mice at middle age (21). However, a direct evidence linking GDF-11 to the pathophysiology of schizophrenia is still lacking.

In view of the vital regulatory role of GDF-11 in normal brain neural development and disrupted brain development in schizophrenia, we hypothesized that abnormal GDF-11 signaling might be implicated with the pathophysiology of schizophrenia. In this study, we verified this hypothesis by examining whether (i) plasma GDF-11 level was changed in schizophrenia patients and (ii) there was any association between GDF-11 level and both psychopathological and cognitive symptoms in these patients.

## Materials and Methods

### Study Subjects

Eighty-seven schizophrenia patients (male/female = 52/35) were enrolled from Jiangxi Mental Hospital, a Nanchang city-owned psychiatric hospital. Two psychiatrists confirmed their diagnosis of schizophrenia based on the Structured Clinical Interviews for DSM-IV Axis I Disorders (SCID). The inclusion criteria included the compliance with DSM-IV diagnostic criteria for schizophrenia, the Positive and Negative Syndrome Scale (PANSS) score ≥ 60, age 18–50 years old, and patients or their families agreed to take part in the research and sign informed consent. Only patients with PANSS total score > 60 were recruited in current study because previous study has demonstrated that being considered “mildly ill” according to the Clinical Global Impressions (CGI) approximately corresponded to a PANSS total score of 61 (22). Furthermore, schizophrenia tends to show up in people around late adolescence or early adulthood (23) and psychosis rarely starts to appear in older aged adults (>50 years of age), thus we recruited patients with age between 18 and 50 years old in this study. The exclusion criteria included patients with severe physical diseases (such as cardiovascular, liver, kidney, gastrointestinal diseases, etc.), infectious diseases and immune system diseases, patients with severe nervous system diseases and mental retardation, patients with other mental illness or drug dependence, and women during pregnancy or lactation. All enrolled patients in this study had not received any antipsychotic medication for at least 3 months before participating in the research. Seventy-six healthy subjects (male/female = 49/27), who were matched with the patients by gender, age, years of education, and body mass index (BMI), were enrolled as control group. Those subjects who presented either a personal or a family history of psychiatric disorders were ruled out from the control group.

The study was conducted in accordance with the Declaration of Helsinki (24) and was approved by the Institutional Review Board at Jiangxi Mental Hospital. All participants or their legal guardians were informed with the content of this study and signed a written informed consent.

### Clinical Assessments

The PANSS (25) was used to evaluate the severity of psychopathology of patients. To ensure consistency and reliability of rating across the study, three psychiatrists participating in this study simultaneously attended a uniform training session in the use of the PANSS prior to the study. After unified training, the correlation analysis showed that the correlation coefficient for the PANSS total score between observers was higher than 0.80.

The Repeatable Battery for the Assessment of Neuropsychological Status (RBANS) (26) was adopted to examine participants' cognitive ability. The scale has good clinical reliability and validity in Chinese population (27). The RBANS consists of 12 subtests that are used to calculate a total score and five age-adjusted index scores. The cognitive domains include Immediate memory (List Learning, Story Memory), Visuospatial/constructional (Figure Copy, Line Orientation), Language (Picture Naming, Semantic Fluency), Attention (Digit Span, Coding), and delayed memory (List Recognition, List Recall, Story Recall, Figure Recall). In this study, the total score and five index scores of RBANS were reported by standard score.

### Plasma GDF-11 Detection

Blood samples were collected from the antecubital vein of patients who were fasted overnight. Whole blood was collected into tubes with EDTA and the tubes were centrifuged at 3,000 rpm for 5 min at temperature of 4°C. The plasma was carefully separated, aliquoted, and stored at −80°C before measurement.

The concentration of GDF-11 was determined by enzyme-linked immunosorbent assay (ELISA). The microplate provided in this kit has been pre-coated with an antibody specific to GDF-11 standards or samples are then added to the appropriate wells with a biotin-conjugated antibody specific to GDF-11. Next, avidin conjugated to Horeradish Peroxidase (HRP) is added to each microplate well and incubated. After tetramethylbenzydine (TMB) substrate solution is added, only those wells that contain GDF-11, biotin-conjugated antibody an enzyme-conjugated avidin will exhibit a change in color. The enzyme-conjugate reaction is terminated by the addition of sulphuric acid solution and the color change is determined by spectrophotometry at a wavelength of 450 ± 10 nm. The detecting O.D. is then compared with the standard curve to determine the concentration of GDF-11 in the sample. The minimum detectable concentration of this method is 6.2 pg/mL. The coefficients of variation for within and between batches are 8 and 11%, respectively.

### Statistical Analysis

Multiple statistical methods were used to compare demographic and clinical variables. Student's *t* test or analysis of variance (ANOVA) were used to statistically analyze continuous variables, and chi-square test were adopted to statistically analyze categorical variables. Since the GDF-11 variables were normally distributed in control and patient groups (the Kolmogorov-Smirnov one-sample test, both *p* > 0.05), one-way ANOVA was adopted to examine where there was a difference in GDF-11 level between two groups. If the ANOVA test displayed a significant result, we continued to use age, gender, years of education, and BMI as covariates to perform the analysis of covariance (ANCOVA) to evaluate the effects of these variables. The Pearson correlation analysis was used to investigate the correlation between variables. Then we conducted partial correlation analysis to determine the associations of GDF-11 levels with psychotic symptoms or cognitive function by adjusting for clinical variables such as gender, age, education years, BMI, duration of illness, as well as age of onset. Bonferroni corrections were conducted to control for multiple testing. Finally, multivariate regression analyses (stepwise regression model) were used to examine the degree of association among independent variables in both patient and control groups. Statistical Product and Service Solutions (SPSS) 22.0 software was used to perform all statistical analyses. Data are presented as mean ± SD. All reported *p*-values are 2-tailed and *p* < 0.05 was considered statistically significant.

## Results

### Demographic and Clinical Characteristics and Cognitive Performance

Table 1 presents the clinical demographic data of healthy controls and schizophrenia patients. There was no significant difference in age, gender, education years, and BMI between two groups (all *p* > 0.05). Table 2 shows the cognitive performances of healthy controls and schizophrenia patients. Patients with schizophrenia performed more poorly in all these cognitive tasks compared to healthy controls (all *p* < 0.05). Significant differences remained after adjusting for age, gender, education years and BMI (all *p* < 0.05).

**Table 1:** Demographic and clinical characteristics between schizophrenia patient group and normal control group.

	**Healthy controls (*n* = 76)**	**Patients with schizophrenia (*n* = 87)**	***F* or χ^2^**	***p***
Sex, M/F	49/27	52/35	0.381	0.537
Age (years)	35.50 ± 8.19	32.98 ± 10.74	2.856	0.093
Education (years)	11.05 ± 2.24	10.74 ± 4.07	0.364	0.547
BMI (kg/m^2^)	22.29 ± 3.9	22.92 ± 3.75	1.315	0.253
Age of onset (years)	–	24.68 ± 6.85	–	–
Duration of illness (years)	–	8.49 ± 7.93	–	–
PANSS total score	–	102.76 ± 8.93	–	–
Positive subscale	–	27.59 ± 6.60	–	–
Negative subscale	–	25.19 ± 6.77	–	–
General psychopathology subscale	–	48.88 ± 11.9	–	–

*M/F, Male/Female; BMI, body mass index; PANSS, Positive and Negative Syndrome Scale*.

**Table 2:** Comparisons of RBANS cognitive function measurements between schizophrenia patient group and normal control group.

	**Healthy controls (*n* = 76)**	**Patients with schizophrenia (*n* = 87)**	***F***	***p***	Adjusted F^*^	P^**^
Immediate memory	77.28 ± 16.623	59.67 ± 12.753	48.506	<0.001	38.819	<0.001
Visuospatial/constructional	79.13 ± 16.324	69.88 ± 14.765	11.693	0.001	9.386	<0.001
Language	81.30 ± 17.390	67.77 ± 17.140	19.931	<0.001	14.725	<0.001
Attention	92.38 ± 15.232	77.18 ± 17.880	26.149	<0.001	10.535	<0.001
Delayed memory	19.83 ± 21.789	65.06 ±16.598	19.999	<0.001	21.102	<0.001
Total score	409.92 ± 69.367	339.57 ± 54.679	43.313	<0.001	33.224	<0.001

* *Adjusted F indicates the F value controlled for age, gender, years of education, and BMI*.

** *p for Adjusted F test*.

*BMI, body mass index; RBANS, Repeatable Battery for the Assessment of Neuropsychological Status*.

### Plasma GDF-11 Levels in Healthy Controls and Schizophrenia Patients

**Figure 1:**
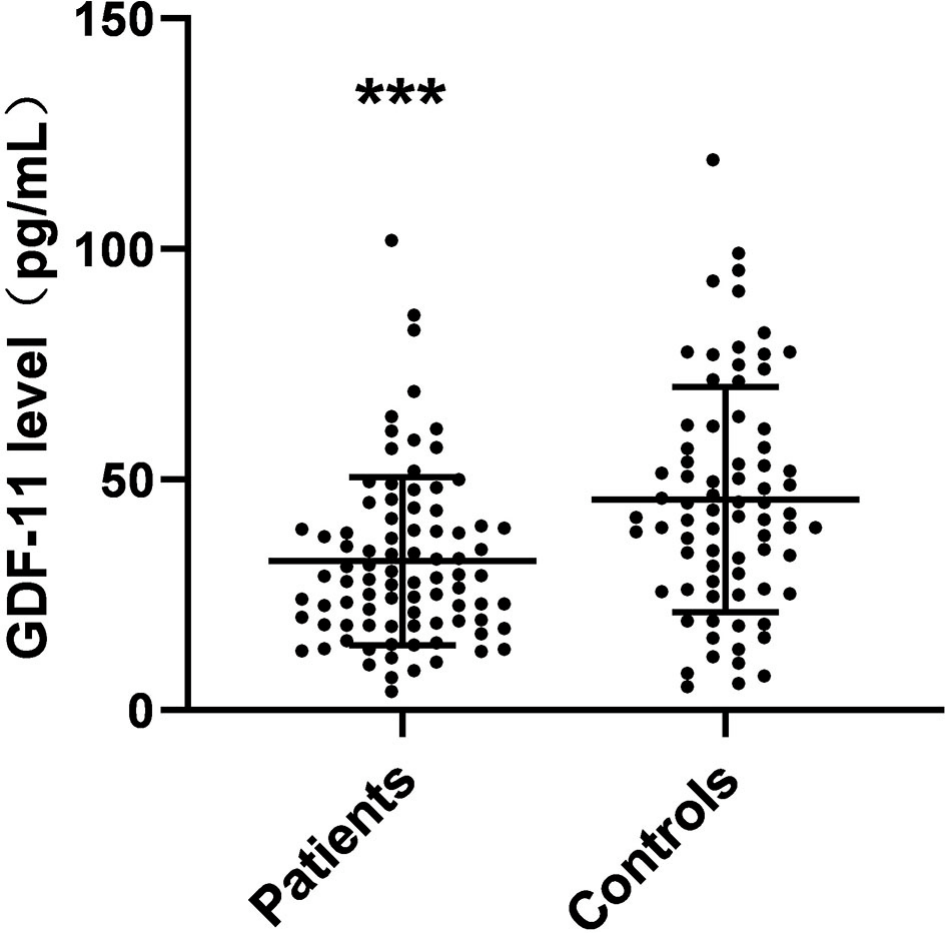
Scattergrams of plasma GDF-11 levels from patients with schizophrenia (*n* = 87) and from normal control subjects (*n* = 76). The sample means are indicated by the black bars. ***p < 0.001.

Plasma GDF-11 levels in control and patient groups are presented in Figure 1. The content of plasma GDF-11 in the patients ranged from 9.91 to 101.94 pg/mL and plasma GDF-11 in the controls ranged from 10.26 to 119.42 pg/mL. Plasma GDF-11 levels were significantly different between patients and controls (32.30 ± 18.25 vs. 45.64 ± 24.41 pg/mL; *F* = 15.87, *p* < 0.001). When age, gender and BMI were used as potentially confounding covariates, the difference between the patient and control groups was still significant (*F* = 4.247, *p* = 0.003).

In the healthy control group, there were no significant correlations between plasma GDF-11 level and age, gender, or BMI, respectively. In the patients, there were no significant correlations between the level of GDF-11 and any demographic variables, including age, gender, BMI, age of psychosis onset, and duration of the illness (all *p* > 0.05).

### Association of GDF-11 Level With Psychopathology in Schizophrenia

**Figure 2:**
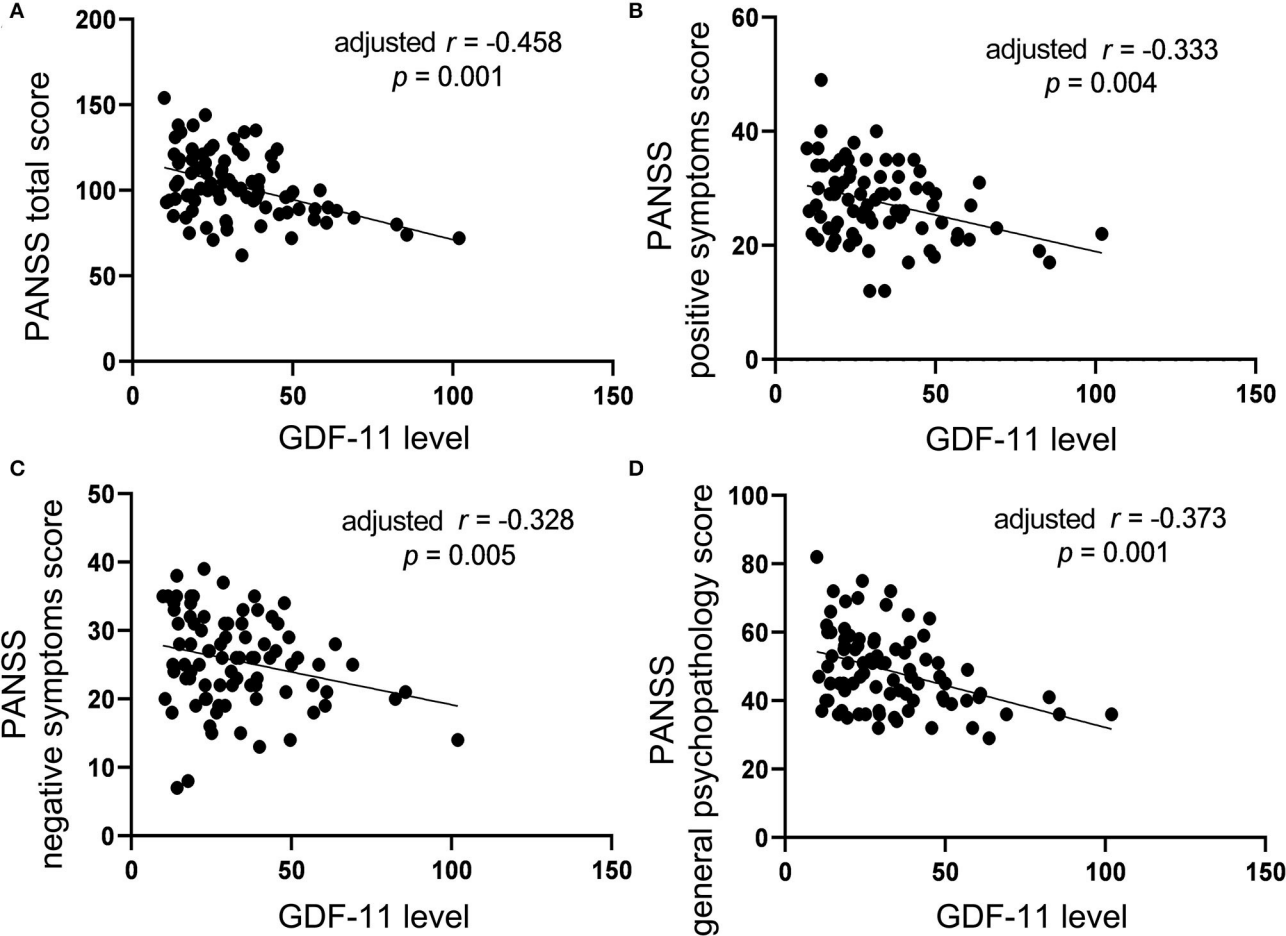
The correlations between plasma GDF-11 level and the PANSS total score **(A)**, the positive symptoms score **(B)**, the negative symptoms score **(C)** or the general psychopathology score **(D)** in patients with schizophrenia. The *r* value in the panel is actually partial correlation coefficient after adjustment for the covariates including age, gender, years of education, BMI, duration of illness, and age of onset.

Correlation analysis showed significant negative associations of plasma levels of GDF-11 with the PANSS total score (*r* = −0.475, *p* < 0.001), the positive symptoms score (*r* = −0.357, *p* = 0.002), the negative symptoms score (*r* = −0.310, *p* = 0.006), or the general psychopathological score (*r* = −0.388, *p* = 0.001) in patients with schizophrenia. Partial correlation analysis demonstrated that the correlations between GDF-11 levels and the PANSS total score (*r* = −0.458, *p* = 0.001), the positive symptoms score (*r* = −0.333, *p* = 0.004), the negative symptoms score *(r* = −0.328, *p* = 0.005), or the general psychopathological score (*r* = −0.373, *p* = 0.001) remained significant after adjusting for gender, age, education years, BMI, duration of illness, and age of onset (Figure 2). Given 9 tests [3 PANSS tests: (i) PANSS positive symptoms score, (ii) PANSS negative symptoms score, (iii) PANSS general psychopathology score, and 6 RBANS tests: (i) RBANS total score, (ii) immediate memory score, (iii) visuospatial/constructional score; (iv) language score; (v) attention score, and (vi) delayed memory score] were performed, multiplicity-adjusted *p*-value threshold based on Bonferroni method is 0.05/9 = 0.005556, and these correlation tests' *p* values remained significant after Bonferroni correction for multiple testing.

### Correlations Between GDF-11 Level and Cognitive Function in Schizophrenia

The RBANS cognitive tests were classified into five domains: immediate memory (List Learning, Story Memory), visuospatial/constructional (Figure Copy, Line Orientation), language (Picture Naming, Semantic Fluency), attention (Digit Span, Coding), and delayed memory (List Recognition, List Recall, Story Recall, Figure Recall). Then the correlations between plasma GDF-11 level and cognitive function in both healthy control and patients were investigated.

**Figure 3:**
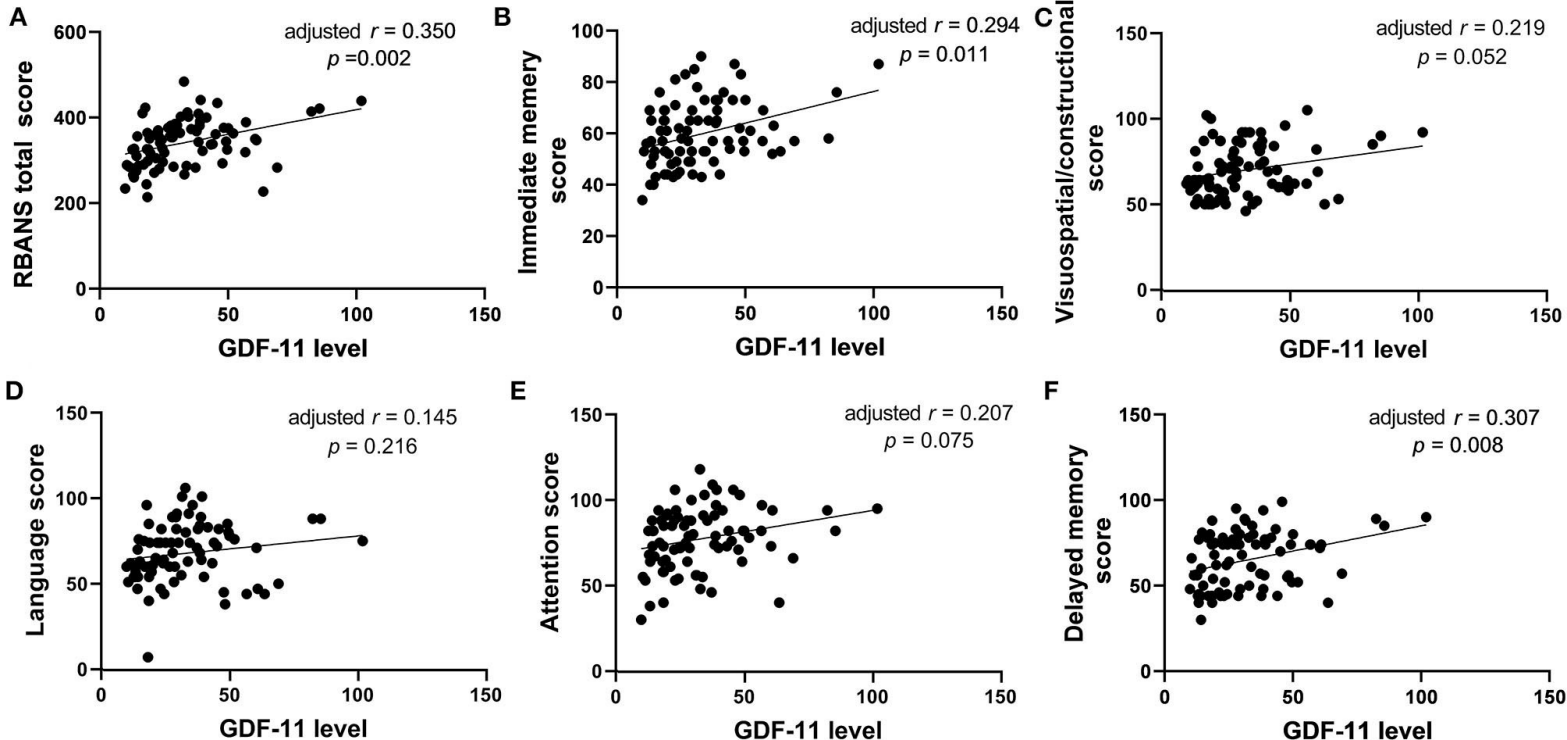
The correlations between plasma GDF-11 level and the RBANS total score **(A)**, immediate memory score **(B)**, visuospatial/constructional score **(C)**, language score **(D)**, attention score **(E)** or delayed memory score **(F)** in patients with schizophrenia. The *r* value in the panel is actually partial correlation coefficient after adjustment for the covariates including age, gender, years of education, BMI, duration of illness, and age of onset.

No significant association was observed between plasma GDF-11 levels and cognitive performances in healthy controls (all *p* > 0.05). For patients, correlation analysis showed significant positive associations of plasma GDF-11 level with the RBANS total score (*r* = 0.383, *p* = 0.001), the immediate memory (*r* = 0.348, *p* = 0.001), the visuospatial/constructional (*r* = 0.249, *p* = 0.023), the attention (*r* = 0.250, *p* = 0.023), or the delayed memory (*r* = 0.383, *p* = 0.001). No significant association was found between plasma GDF-11 level and the language index (*r* = 0.159, *p* = 0.152). Partial correlation analysis showed that the correlations between GDF-11 level and the total score (*r* = 0.350, *p* = 0.002), the immediate memory (*r* = 0.294, *p* = 0.011) or the delayed memory (*r* = 0.307, *p* = 0.008) were still significant after adjusting for gender, age, education years, BMI, duration of illness, and age of onset (Figure 3). Given 9 tests [3 PANSS tests: (i) PANSS positive symptoms score, (ii) PANSS negative symptoms score, (iii) PANSS general psychopathology score, and 6 RBANS tests: (i) RBANS total score, (ii) immediate memory score, (iii) visuospatial/constructional score; (iv) language score; (v) attention score, and (vi) delayed memory score] were performed, multiplicity-adjusted *p*-value threshold based on Bonferroni method is 0.05/9 = 0.005556, and these correlation tests' *p* values remained significant after Bonferroni correction for multiple testing.

Then we performed multiple regression analysis to examine independent determinants of RBANS and its indexes. For the healthy controls, GDF-11 was not a contributor to any of the total or sub-index score of RBANS (all *p* > 0.05). For the patients, GDF-11 was an independent contributor to the immediate memory (*t* = 3.334, *p* = 0.001), delayed memory (*t* = 3.036, *p* = 0.003) and RBANS total score (*t* = 3.371, *p* < 0.001). PANSS negative symptoms score was an independent contributor to the visuospatial/constructional (*t* = −2.552, *p* = 0.013), and PANSS total score was an independent contributor to the attention index of RBANS (*t* = −3.524, *p* = 0.001).

## Discussion

Our study revealed that (i) plasma GDF-11 levels were significantly lower in schizophrenia patients than those in healthy controls; (ii) there were significant negative correlations between GDF-11 level and the PANSS total score, positive symptoms score, negative symptoms score or general psychopathological score in patients; and (iii) plasma GDF-11 level was positively associated with the immediate memory and delayed memory in patients. For all we know, this study is the first to show that GDF-11 is associated with the psychopathology and cognition in schizophrenia.

GDF-11 is a member of the transforming growth factor β superfamily (28). It is widely expressed in adult CNS and has a crucial impact on brain development (11). Previous study has shown that GDF-11 can enhance neurogenesis in the brain, especially in the hippocampus, helping remodeling the synapses and repairing the injured neurons (15, 29, 30). It can also regulate the function of astrocytes, whose activation are shown to contribute to the pathogenesis of schizophrenia (11). Our present findings show a significant decrease in plasma GDF-11 levels in schizophrenia patients, and GDF-11 level was related to the psychopathological symptoms and cognitive impairments. In combination with the roles of GDF-11 in neurogenesis and brain function (15, 29, 31) and the effects of GDF-11 on nerve development and cell regulations (32–34), it is indicated that GDF-11 may be related to the pathophysiology of schizophrenia. However, it should be noted that the difference in plasma GDF-11 levels between the patient and control groups could result from schizophrenia itself or other potential influence factors, such as the effect of antipsychotics. In this study, we enrolled patients who had not received any antipsychotic medication at least for 3 months before participating in the research. Combined with the findings that the decrease of plasma GDF-11 level was correlated to the severity of psychotic symptoms and cognitive impairments, we speculate that the population difference of plasma GDF-11 is more likely to be from to the disease itself, rather than to the secondary influence of drug therapy.

There were significantly negative relationships between GDF-11 level and the PANSS total score, or the positive, negative, and general psychopathological subscore, suggesting that patient with lower GDF-11 level would have a more severer psychopathological symptoms. Many studies could support the presumption that decrease of GDF-11 in schizophrenia might not be just an incidental phenomenon, but be associated to the pathological mechanisms of schizophrenia. Specifically, patients with abnormal brain function and structure such as Alzheimer's disease (35), Parkinson's syndrome (36) or craniocerebral trauma (37) show psychotic symptoms such as delusions and emotional disorders. Treatment with GDF-11 can effectively improve the symptoms of Alzheimer's and stroke patients (29, 38). The study of Katsimpardi et al. has also demonstrated that GDF-11 can improve the cerebral vasculature and enhance the neurogenesis (20). Systemic GDF-11 treatment significantly improved the vasculature in the cortex and hippocampus of old mice (15). However, this presumption is still required to be tested by using animal experiments. Furthermore, the exact mechanism of the decrease in GDF-11 levels in patients with schizophrenia is unclear, requiring further study.

Cognitive impairments are generally deemed as a core symptom of schizophrenia (39). A large number of studies have reported that patients with schizophrenia have cognitive impairments in many aspects, including speech learning, visual memory, attention, processing speed, working memory, and executive function (40, 41). Our results showed that the performances of immediate memory, language, attention, visuospatial/constructional and delayed memory in patients with schizophrenia were much poorer than those in the normal control group, and GDF-11 was positively associated with immediate memory and delayed memory in schizophrenia patients. GDF-11 can regulate the neuronal neurodevelopment process, including induction, proliferation, migration and survival of cells (15, 33, 34). Furthermore, GDF-11 is shown to have novel roles in neuronal plasticity and cognition (15), in which GDF-11 promotes synaptic remodeling in the hippocampus (20). Thus, the finding that decreased plasma GDF-11 levels were correlated with cognitive impairments in patients with schizophrenia suggests that abnormal GDF-11 signaling is implicated with the pathogenesis of schizophrenia-associated cognitive impairments. However, it is uncertain whether the decline in GDF-11 levels in patients with schizophrenia is accidental or related to the pathogenesis of schizophrenia, as this is a cross-sectional study at which other factors, including the secondary influences of drug therapy and chronic diseases, cannot be completely excluded. More researches including animal experiments or longitudinal and prospective studies are required to further test these hypotheses.

The neurodevelopmental hypothesis of schizophrenia holds that the function state of the nervous system would be disturbed by genetic and early environmental factors during postnatal development, leading to abnormal neuronal proliferation and differentiation, excessive pruning, abnormal synaptic connection (4, 42). GDF-11 can enhance neurogenesis in the brain, helping remodeling the synapses and repairing the injured neurons (43). It also regulates the function of astrocytes and brain activity such as the dopamine neural loop (44), whose dysfunctions would cause schizophrenia-associated behavioral and cognitive symptoms. In this study, we showed a significant decrease in plasma GDF-11 level in schizophrenia, and decreased GDF-11 was correlated with the psychopathology and cognitive function of patients. Therefore, we postulate that disrupted GDF-11 signaling results in abnormalities of neurogenesis, astrocyte function and brain activity during early development, leading to the emergence of psychotic symptoms and cognitive disorders in late adolescence and young adulthood.

Our research has some limitations. First of all, although we found that decreased plasma GDF-11 was closely related to the severities of psychopathology and cognitive impairments in schizophrenia patients, the exact mechanism of GDF-11 affecting schizophrenia-related behaviors is not clear. It is necessary to conduct animal experiments to further reveal the mechanism of GDF-11 and its signaling pathway. Second, we only measured the level of GDF-11 in plasma, not the level of GDF-11 in the cerebrospinal fluid. This is not enough to infer similar changes in GDF-11 in the CNS and peripheral nervous system. Third, the number of schizophrenia subjects was relatively small in the present study. Larger-scale clinical research is necessary to confirm this finding in the future. Finally, all study subjects were of Chinese ancestry, which could limit the generalizability of the findings to other ethnic groups.

Taken together, our study revealed that decreased plasma GDF-11 in Chinese patients with schizophrenia was significantly correlated with the patients' psychopathological and cognitive symptoms, suggesting that altered GDF-11 signaling might contribute to the psychopathology and cognitive deficits in schizophrenia.

## Data Availability Statement

The raw data supporting the conclusions of this article will be made available by the authors, without undue reservation.

## Ethics Statement

The studies involving human participants were reviewed and approved by Institutional Review Board at Jiangxi Mental Hospital. The patients/participants provided their written informed consent to participate in this study.

## Author Contributions

Z-xY, J-qZ, J-wX, BW, Y-hF, Z-pL, Y-tT, and A-lW participated in clinical data collection and lab data analysis. Y-jY and Z-xY designed the study, analyzed data, and prepared the manuscript. All authors contributed to the article and approved the submitted version.

## Conflict of Interest

The authors declare that the research was conducted in the absence of any commercial or financial relationships that could be construed as a potential conflict of interest.
